# The Healthy Lifestyle and Personal Control Questionnaire (HLPCQ): a novel tool for assessing self-empowerment through a constellation of daily activities

**DOI:** 10.1186/1471-2458-14-995

**Published:** 2014-09-24

**Authors:** Christina Darviri, Evangelos C Alexopoulos, Artemios K Artemiadis, Xanthi Tigani, Christina Kraniotou, Panagiota Darvyri, George P Chrousos

**Affiliations:** Postgraduate Course Stress Management and Health Promotion, School of Medicine, University of Athens, Soranou Ephessiou 4, GR-11527 Athens, Greece; First Department of Pediatrics, Children’s Hospital Aghia Sofia, School of Medicine, University of Athens, Thivon & Papadiamantopoulou Str, GR-115-27 Athens, Greece

**Keywords:** Health, Lifestyle, Wellbeing, Empowerment, Daily routine

## Abstract

**Background:**

The main goal of stress management and health promotion programs is to improve health by empowering people to take control over their lives. Daily health-related lifestyle choices are integral targets of these interventions and critical to evaluating their efficacy. To date, concepts such as self-efficacy, self-control and empowerment are assessed by tools that only partially address daily lifestyle choices. The aim of this study is to validate a novel measurement tool, the Healthy Lifestyle and Personal Control Questionnaire (HLPCQ), which aims to assess the concept of empowerment through a constellation of daily activities.

**Methods:**

Therefore, we performed principal component analysis (PCA) of 26 items that were derived from the qualitative data of several stress management programs conducted by our research team.

**Results:**

The PCA resulted in the following five-factor solution: 1) Dietary Healthy Choices, 2) Dietary Harm Avoidance, 3) Daily Routine, 4) Organized Physical Exercise and 5) Social and Mental Balance. All subscales showed satisfactory internal consistency and variance, relative to theoretical score ranges. Subscale scores and the total score were significantly correlated with perceived stress and health locus of control, implying good criterion validity. Associations with sociodemographic data and other variables, such as sleep quality and health assessments, were also found.

**Conclusions:**

The HLPCQ is a good tool for assessing the efficacy of future health-promoting interventions to improve individuals’ lifestyle and wellbeing.

## Background

To date, numerous environmental health-related lifestyle factors have been extensively studied, such as dietary habits, substance abuse (e.g., smoking), physical exercise, and sleeping quality [1]. The hallmark of this vast literature is “lifestyle choices”, which suggests that all these factors are amenable to change. As a consequence, health professionals, stakeholders, health organizations, and even governments, in different time periods and countries, have implemented numerous health promotion programs targeting either specific health-related risk factors (e.g., smoking) or disorders (e.g., cardiovascular diseases). The effectiveness of these programs has been broadly questioned because they do not seem to intercept the gradual rise of chronic diseases, especially in developing countries [2, 3]. Analysis of this putative failure is beyond the scope of this article, although we can deduce that these programs presumably fail to empower and achieve behavioral change among individuals [4, 5].

Health empowerment suggests that the individual has increased control over his/her life and health or has an internal health locus of control. This may seem particularly difficult within modern, hectic environments that are the source of numerous stressors (e.g., work demands, family breeding, transportation etc.) that threaten the individual. The interplay between stress and self-control has long concerned the scientific community. In summary, chronic or extreme stress has detrimental effects on daily decision making by favoring choices of immediate physical reward in an effort to relieve emotional distress, thus creating a vicious cycle of adversity. However, “healthy” choices reflect an empowered individual, capable of controlling stress and making decisions that have long-term benefits (For a comprehensive review, see ref [6]). These concepts may also extend to the field of health promotion that strives to empower people to make healthy lifestyle choices that will prevent chronic disease and morbidity.

Thus, stress management may facilitate health promotion and its primary goal of empowering individuals. In our department, we have conducted several investigations in this field, primarily by implementing stress management programs in different subsets of the population (healthy or not) ([7–13] and unpublished studies). In our experience, which included personal contact with participants and collection of qualitative data, stress management/health promotion interventions resulted in additional lifestyle modifications of diet, exercise, social support and daily routine that were not detected by our studies’ main measurements tools (i.e. stress scales, health locus of control scales, self-esteem etc.).

Therefore, we have designed a short questionnaire that synthesizes items related to lifestyle changes and is based on our experience with stress management research. The novel idea behind this tool is that it does not simply record common health-related daily activities, but it measures the isolated behaviors of well-proven health-related aspects of daily living to evaluate the degree of control that someone exerts on his/her life. Although, researchers prefer to assess empowerment, self-control or self-efficacy with homonymous questionnaires [14–16], this tool obviates the need for theoretical items (“How capable are you of controlling your life?”) that sometimes confuse participants and furthers such evaluations by simply inspecting various aspects of the individual’s life.

The aim of this study is to examine the psychometric properties of this tool. Cross-validation (criterion validity) of this instrument was based primarily on perceived stress and health locus of control (representing empowerment) questionnaires.

## Methods

The study was performed in the province of Attica, Greece, between November 2011 and January 2012. The study did not require approval according to the Scientific and Ethics committee of Athens’s Medical University. Our study was a convenience sample of 308 individuals who were asked to participate in the study; 28 of these were postgraduate medical school students and 280 were friends and/or relatives of the former. After being fully informed of the purposes of the study and giving their consent, the participants were asked to complete an anonymous questionnaire. Inclusion criteria were the ability to read and write in Greek, 20–80 years of age and residency in Attica. Finally, 285 participants (92.5% response rate) delivered a completed questionnaire. This manuscript adheres to RATS reporting guidelines.

### The Health Lifestyle and Personal Control Questionnaire (HLPCQ)

This is a 26-item tool in which the respondent is asked to indicate the frequency of adopting 26 positively stated lifestyle habits using a Likert-type scale (1 = Never or rarely, 2 = Sometimes, 3 = Often and 4 = Always). The introductory phrase is “How often…” There are 12 items concerning diet, 8 items referring to a daily time management, 2 items referring to organized physical exercise and 4 items referring to practices of social support and positive thinking (e.g., positive thoughts during difficulties and emptying the mind during bedtime). As stated above, items were derived from our experience with stress management/health promotion interventions used in different study populations. In the end of each intervention program participants were asked about lifestyle changes that they have noticed during the previous weeks, using the following open question: “During the previous weeks, have you noticed any changes concerning your everyday living/lifestyle?”. The 26 items presented in the HLPCQ questionnaire are the result of gathering all the qualitative data from the participants’ answers (a total of 305 participants) to the aforementioned open question. All answers without exceptions were grouped and rephrased to keep the initial participant’s meaning. Our main goal for this questionnaire was to detect and quantify lifestyle patterns that reflect health empowerment, as evidenced by the levels of stress and of the internal health locus of control. As such, validation is based upon these two characteristics, perceived stress and health locus of control, using the questionnaires described below.

### Other measurements

#### Sociodemographic variables included

Gender, age, marital status (married/unmarried), domestic status (living alone or not), presence of children, education (tertiary - above 12 education years -/secondary - 6–12 education years- or lower), employment (employed/retired/household/unemployed), working shifts (yes/no), care-giving (yes/no), smoking (yes/no), pack-years of smokers, body mass index (Kg/m^2^) and presence of disease. Questions used to collect information about these variables have been previously described [17, 18].

#### Perceived Stress Scale (PSS)

The PSS is a self-reported 14-item measure indicating the degree to which situations in an individual’s life are considered stressful [19]. For this scale, respondents rate the frequency of their feelings and thoughts over the previous month on a five-point Likert-type scale (from 0 = never to 4 = very often). There are seven positive and seven negative items, and the total score is calculated by reversing the scores of the positive items and then summing all scores (min. total score = 0, max. total score = 56). Higher scores indicate higher level of perceived stress. Good psychometric properties of this measure within the Greek population have been reported [20]. In addition, the internal consistency of this 14-item scale in this study was also good (Cronbach’s alpha, 0.85).

#### Social Readjustment Rating Scale

Life events that occurred more than 12 months prior to the survey were assessed using the Holmes Rahe Social Readjustment Rating Scale [21, 22]. Participants were asked about 43 life events that are thought to induce change in an individual’s life. Life event data were then summarized in accordance with SRRS scoring rules. Each life event was assigned a predetermined number of life change units ranging from 11 to 100. Life change units were then summed for each participant to calculate a total SRRS score; higher SRRS scores indicated greater stress. SRRS scores were categorized as low (<150), medium (150–299), or high (≥300) [21, 22].

#### Health Locus of Control (HLC)

Health locus of control was measured using the 18-item tool developed by Wallston and colleagues [23]. The respondents expressed their level of agreement to 18 statements on a 6-point Likert-type scale (from 1 = strongly disagree to 6 = strongly agree). The scale is built upon three 6-item subscales, namely: “internal health locus of control”, “external health locus of control” and “chance”. The “internal health locus of control” measures the degree to which the individual believes that he/she is responsible for his/her health status. The “external health locus of control” and “chance” represent the extent to which other people (such as physicians) or chance, respectively, are deemed important to the individual’s health. After summing the answers for each subscale, higher scores indicate higher strength of each type of health belief (total score range was 6–36 for each subscale). The instrument has been applied to Greek samples [24]. The internal consistency for each subscale was found to be satisfactory (Cronbach’s alphas: “internal” 0.72, “external” 0.75 and “chance” 0.71).

#### Sleep Quality (SQ)

Sleep quality was assessed using the following questions: 1. “Are you satisfied with your sleep?” (Answers: 0 = Not at all, 1 = Little, 2 = Moderate, 3 = Very, 4 = Very much), 2. “Do you take any drug in order to sleep?” (Answers: −1 = Yes, 0 = No), 3. “Do you fall asleep easily?” (Answers: 0 = Never, 1 = Sometimes, 2 = Often, 3 = Always) and 4. “Do you feel restful after awakening?” (Answers: 0 = Never, 1 = Sometimes, 2 = Often, 3 = Always). The total score is calculated by summing all answers (minimum −1, maximum 10); higher scores indicate better sleep quality.

#### Health Assessment (HA)

Each individual was asked to rate his/her health on a scale from 1 to 10, in which 10 denoted excellent health.

### Statistical analyses

Descriptive analyses were used to calculate the means, standard deviations (SD), minimums, maximums and absolute and relative frequencies (%). Principal component analysis (PCA) was used to identify the factors from the HLPCQ. Bartlett’s test was used to assess whether the correlation between items was adequate; in contrast, a determinant value was calculated to assess unwanted over-correlation of items (determinant should be close to zero). The Kaiser-Meyer-Olkin (KMO) statistic was used to assess sample adequacy. The appropriate number of derived factors were identified using the scree-plot (looking for inflexion points) and Kaiser’s criterion of eigenvalues greater than 1 (given that our sample was large, the criterion is valid for an average of communalities greater than 0.6). Loadings of each item on derived factors were maximized using the orthogonal varimax rotation. Items with loadings above 0.3 were examined as candidate components of the corresponding factor. Cronbach’s alpha values were calculated to assess internal consistency of the identified factors. After, the scores of each factor were calculated and assessed for meaningful associations with the other measurements of the study. For group comparisons, we used Student’s *t*-test, and for scale variables, we used Pearson’s rho correlation coefficient. The level of significance *p* was *.*05. Statistical analyses were performed using the SPSS for Windows (version 18.0.3) statistical software (SPSS Inc., Chicago, IL).

## Results

Table 1 presents the main characteristics of our sample. A majority of participants were middle-aged (from 23 to 76 years old), married with children, women of tertiary education and currently employed (11 unemployed, 30 household, 30 retired and 214 employed). In addition, 55 individuals were living alone at home, 64 (22.5%) had a job with working shifts and 22 (7.7%) were caregivers of handicapped patients. Additionally, 28% of our sample were smokers with a mean history of 24.82 pack-years. The mean BMI was 26.34 Kg/m^2^ (minimum 21.8 Kg/m^2^, maximum 51.9 Kg/m^2^, 121 (42.5%) persons had a BMI ≤25 Kg/m^2^), indicating an overweight problem. In addition, 130 (45.6%) individuals reported a diagnoses of disease by their physician. For completeness, descriptive statistics for the PSS, SRRS, HLC, SQ and HA are also presented.

**Table 1:** **Sociodemographic and health-related characteristics of the study’s sample (N = 285)**

***Sociodemographic characteristics***		***Health-related characteristics***	
Males N (%)	119 (41.8)	Smokers N (%)	81 (28.4)
Mean Age in years (SD)	50.18 (10.1)	Mean Pack-years for smokers (SD)	24.82 (21.8)
Married N (%)	188 (66)	Mean BMI in Kg/m^2^ (SD)	26.34 (5.34)
Living alone N (%)	55 (19.3)	Diagnosis of Disease N (%)	130 (45.6)
Having children N (%)	209 (73.3)	Mean PSS score (SD)	26.35 (7.99)
Tertiary education N (%)	165 (57.9)	Mean Internal HLC score (SD)	26.12 (4.96)
Employed N (%)	214 (75.1)	Mean External HLC score (SD)	22.23 (6.04)
Working shifts N (%)	64 (22.5)	Mean Chance HLC score (SD)	16.75 (6.04)
Caregivers N (%)	22 (7.7)	SRRS score N (%)	
		≤150	164 (57.5)
		151-299	80 (28.1)
		≥300	41 (14.4)
		Mean Sleep Quality (SD)	6.16 (2.13)
		Mean Health Assessment (in a scale from 1 to 10) (SD)	7.48 (1.4)

SD: standard deviation, BMI: Body Mass Index, PSS: Perceived Stress Scale, HLC: Health Locus of Control, SRRS: Social Readjustment Rating Scale.

The results of the principal component analysis (PCA) of the 26 items with orthogonal rotation (varimax) are presented in Table 2. The Kaiser-Meyer-Olkin measure verified the sampling adequacy for the analysis (KMO = .797) and all KMO measures for individual items were > .603, which is well above the acceptable limit of .5. Bartlett’s test of sphericity *x*^2^(325) = 1903.33, *p < .001*, indicated that correlations between items were sufficiently large enough to perform PCA. The determinant was zero indicating lack of excessive correlations between items. Seven components had eigenvalues greater than Kaiser’s criterion of 1 and in combination explained 58.58% of the variance. The average of communalities was .58, which is below the Kaiser’s criterion of .6 to be accurate. The scree-plot (not shown) supported the choice for the selection of five components according to the inspection of inflexion points, which explained 49.69% of the variance. Given the large sample size, five components were retained for final analysis. The clusters of items, according to factor loadings (>0.3), within the five components were interpreted as following: “Dietary Healthy Choices” (DHC), “Dietary Harm Avoidance” (DHA), “Daily Routine” (DR), “Organized Physical Exercise” (OPE), and “Social and Mental Balance” (SaMB).

**Table 2:** **Rotated factor loadings of the principal components analysis (PCA) for 26 health-related lifestyle habits (N = 285)**

Item “How often…”	“Dietary Healthy Choices”	“Dietary Harm Avoidance”	“Daily Routine”	“Organized Physical Exercise”	'Social and Mental balance”
“Are you careful about how much food you put on your plate”	.51				
“Do you check the food labels before buying a product”	.51				
“Do you calculate the calories of your meals”	.51				
“Do you limit fat in your meals”	.53				
“Do you like cooking”	.59				
“Do you eat organic foods”	.65				
“Do you eat whole-wheat products”	.63				
“Do you avoid eating packaged- or fast-food”		.67			
“Do you avoid soft drinks”		.59			
“Do you avoid eating when stressed or disappointed”		.68			
“Do you avoid binge eating when you are out with friends”		.64			
“Do you eat your meals at the same time each day”			.67		
“Are you careful about not missing a meal each day”			.6		
“Do you eat a good breakfast”			.58		
“Do you sleep at the same time each day”			.6		
“Do you follow a scheduled program for your daily activities”			.35		
“Do you eat breakfast at the same time each day”			.74		
“Do you eat lunch at the same time each day”			.73		
“Do you eat dinner at the same time each day”			.7		
“Do you practice aerobic exercise for 20 or more minutes at least 3 times per week				.83	
“Do you exercise in an organized manner”				.84	
“Do you share your personal problems or worries with others”					.61
“Do you concentrate on positive thoughts during difficulties”					.57
“Do you empty your brain of thoughts or the next day’s program during bedtime”					.53
“Do you care about meeting and discussing with your family on a daily basis”					.73
“Do you balance your time between work, personal life and leisure”					.53
**Eigenvalues**	2.79	2.42	3.54	1.99	2.18
**% of Variance**	10.75	9.31	13.59	7.66	8.38
**Cronbach’s alpha**	.748	.651	.818	.782	.627

**Important Note:** The translation of the items from the Greek language is presented only for interpretation and NOT for use in studies or clinical practice.

Table 3 presents the mean scores of each subscale along with the theoretical and observed values of the range. It is evident that there was a good dispersion of calculated scores in our sample relative to the possible range of scores.

**Table 3:** **Descriptive characteristics of the five subscales and the total score of HLPCQ**

	Items	Range	Mean	SD	Minimum	Maximum
“Dietary Healthy Choices”	7	7-28	15.81	4.43	7	28
“Dietary Harm Avoidance”	4	4-16	10.32	2.86	4	16
“Daily Routine”	8	8-32	21.75	5.14	10	32
“Organized Physical Exercise”	2	2-8	3.84	2.01	2	8
“Social and Mental Balance”	5	5-20	12.94	2.95	5	20
Total Score	26	26-104	64.61	11.84	35	98

HLPCQ: Healthy Lifestyle and Personal Control Questionnaire, SD: Standard Deviation.

Table 4 presents the correlations between subscales. Specifically, all subscales were significantly positively correlated with each other, indicating that individuals adopting healthy dietary habits and avoiding dietary harms also follow a daily routine in their activities, exercise in an organized manner, seek for social support and care for their mental health.

**Table 4:** **Correlations (Pearson’s rho) between HLPCQ subscales**

	“Dietary Healthy Choices”	“Dietary Harm Avoidance”	“Daily Routine”	“Organized Physical Exercise”	“Social and Mental Balance”
“Dietary Healthy Choices”	1				
“Dietary Harm Avoidance”	.398*	1			
“Daily Routine”	.414*	.337*	1		
“Organized Physical Exercise”	.307*	.231*	.253*	1	
“Social and Mental Balance”	.387*	.202*	.343*	.208*	1

*Correlation is significant at the 0.05 level (2-tailed).

HLPCQ: Healthy Lifestyle and Personal Control Questionnaire.

Tables 5 and 6 present meaningful associations between the HLPCQ subscales and the total score and study variables. Significant associations with the subscales and the total score can be summarized as follows: 1. Women have significantly higher DHC, SaMB and total HLPCQ scores than men, 2. Younger (less than 46 years old) individuals practice organized exercise more frequently than do older ones, 3. Unmarried people seek social support and care for their mental health more frequently than married ones, and they have higher total HLPCQ scores, 4. People with tertiary education have significantly greater DHC and OPE scores than those with lower education, 5. Non-smokers have higher scores in all HLPCQ subscales except the SaMB subscale and in the total score than smokers, and heavier smoking (as expressed by pack-years) was significantly correlated with lower DHC and SaMB scores 6. As expected, a higher BMI was correlated with significantly less exercise, as indicated by lower OPE scores and lower HLPCQ scores. 7. Better health assessment was significantly correlated with higher scores in all subscales and the total score, 8. A lower PSS score was significantly correlated with higher scores in all subscales and the total HLPCQ score, 9. Internal HLC was significantly positively correlated with DHC, DHA, OPE, SaMB and the HLPCQ score, 10. External HLC was negatively correlated only with OPE, 11. Chance HLC was negatively correlated with the subscales DHC, DHA, OPE, and SaMB and with the total HLPCQ score and 12. Sleep quality was positively correlated with DHC, DR, OPE, and SaMB and the total HLPCQ score.

**Table 5:** **Association between HLPCQ subscales and other study measurements**

Characteristics	Categories	Mean “Dietary Healthy Choices-DHC” (SD)	Mean “Dieatary Harm Avoidance-DHA” (SD)	Mean “Daily Routine-DR” (SD)	Mean “Organized Physical Exercise-OPE” (SD)	Mean “Social and Mental Balance-SaMB” (SD)
**Gender**	Males	14.16 (4.1)	9.97 (2.85)	21.79 (5.03)	3.71 (2.01)	12.44 (2.770
	Females	17 (4.32)	10.59 (2.86)	21.76 (5.23)	3.93 (2.02)	13.33 (3.03)
	Statistics	t(277) = 5.54	t(276) = 1.78	t(252) = 0.05	t(282) = 0.91	t(279) = 2.52
	*p value*	<.001*	*0.08*	*0.96*	*0.36*	0.01*
**Age categories**	≤46	15.63 (4.13)	10.17 (2.55)	21.3 (5.13)	4.32 (2.03)	13.05 (3.0)
	47-55	15.76 (4.72)	10.1 (2.88)	21.7 (5.11)	3.4 (1.77)	12.98 (3.01)
	≥56	16.1 (4.42)	10.77 (3.15)	22.29 (5.21)	3.84 (2.01)	12.76 (2.84)
	Statistics	F(2,277) = 0.23	F(2,246) = 1.47	F(2,252) = 0.76	F(2,282) = 5.41	F(2,278) = 0.23
	*p value*	*.8*	*.23*	*.47*	.01*	*.8*
**Marital Status**	Unmarried	16.11 (4.64)	10.23 (2.64)	22.52 (4.71)	4.03 (2.1)	13.44 (2.96)
	Married	15.66 (4.32)	10.37 (2.98)	21.31 (5.34)	3.73 (1.96)	12.68 (2.92)
	Statistics	t(278) = 0.8	t(277) = 0.38	t(253) = 1.82	t(283) = 1.18	t(280) = 2.09
	*p value*	*.43*	*.7*	*.07*	*.24*	.04*
**Education**	Sercondary or lower	15.19 (4.36)	10.33 (3.05)	21.39 (5.61)	3.35 (1.85)	12.89 (2.88)
	Tertiary	16.26 (4.43)	10.31 (2.73)	21.99 (4.8)	4.19 (2.06)	12.99 (3.01)
	Statistics	t(278) = 2.01	t(277) = 0.05	t(253) = 0.9	t(283) = 3.54	t(280) = 0.24
	*p value*	.046*	*.96*	*.37*	<.001*	*.81*
**Working Shifts**	No	15.31 (4.28)	10.23 (2.730	22.06 (5.07)	3.89 (2.1)	12.78 (2.71)
	Yes	16.45 (5.24)	10.21 (3.12)	21.82 (5.27)	3.64 (1.86)	13.37 (3.31)
	Statistics	t(100) = 1.55	t(212) = 0.05	t(191) = 0.29	t(217) = 0.83	t(97.6) = 1.24
	*p value*	*.13*	*.96*	*.77*	*.41*	*.22*
**Smoking**	Yes	14.29 (4.39)	9.41 (2.84)	19.66 (5.31)	3.32 (1.89)	12.46 (3.23)
	No	16.35 (4.24)	10.65 (2.78)	22.55 (4.86)	4.02 (2.02)	13.11 (2.82)
	Statistics	t(277) = 3.62	t(276) = 3.32	t(253) = 4.14	t(282) = 2.7	t(279) = 1.68
	*p value*	<.001*	*.001*	<.001*	.01*	*.09*
**Disease**	Yes	15.76 (4.43)	10.32 (2.98)	22.17 (5.26)	3.71 (1.93)	12.61 (3.06)
	No	15.84 (4.44)	10.32 (2.77)	21.37 (5.03)	3.94 (2.08)	13.21 (2.83)
	Statistics	t(278) = 0.16	t(277) = 0.02	t(253) = 1.25	t(283) = 0.98	t(280) = 1.72
	*p value*	*.87*	*.99*	*.21*	*.33*	*.09*
**Packet-years for smokers**	Pearson’s rho	-.23	.01	-.09	-.19	-.24
	*p value*	.046*	*.92*	*.45*	*.08*	.03*
**BMI (kg/m** ^**2**^ **)**	Pearson’s rho	-.09	-.06	-.09	-.24	-.04
	*p value*	*.13*	*.27*	*.15*	<.001*	*.53*
**Health Assessemnt (in a scale from 1 to 10)**	Pearson’s rho	.15	.17	.17	.23	.25
	*p value*	.01*	.01*	.01*	<.001*	<.001*
**SRRS score**	Pearson’s rho	.03	-.03	-.08	.11	-.03
	*p value*	*.61*	*.58*	*.22*	*.06*	*.68*
**PSS score**	Pearson’s rho	-.29	-.27	-.26	-.25	-.38
	*p value*	<.001*	<.001*	<.001*	<.001*	<.001*
**Internal HLC**	Pearson’s rho	.17	.12	.05	.14	.25
	*p value*	.004*	.04*	*.41*	.02*	<.001*
**External HLC**	Pearson’s rho	-.02	.01	-.02	-.15	.11
	*p value*	*.71*	*.82*	*.82*	.02*	*.07*
**Chance HLC**	Pearson’s rho	-.14	-.12	-.1	-.14	-.25
	*p value*	.03*	.04*	.11	.02*	<.001*
**Sleep quality**	Pearson’s rho	.2	.08	.22	.17	.36
	*p value*	.001*	*.22*	.001*	.004*	<.001*

*Level of significance <0.05.

**Table 6:** **Associations between HLPQ total score and other study measurements**

Characteristics	Categories	Mean “Healthy Lifestyle Questionnaire”	Characteristics	Correlations	“Healthy Lifestyle Questionnaire”
**Gender**	Males	62 (10.8)	**Packet-years for smokers**	Pearson’s rho	-.24
	Females	66.52 (12.2)		*p value*	.06
	Statistics	t(242) = 2.99	**BMI (kg/m** ^**2**^ **)**	Pearson’s rho	-.15
	*p value*	.003*		*p value*	.02*
**Age Categories**	≤46	64.4 (11.62)	**Health Assessment (in a scale from 1 to 10)**	Pearson’s rho	.28
	47-55	63.9 (11.62)		*p value*	<.001*
	≥56	65.73 (13.05)	**SRRS score**	Pearson’s rho	.02
	Statistics	F(2,242)		*p value*	.8
	*p value*	*.61*	**PSS score**	Pearson’s rho	-.42
**Marital Status**	Unmarried	66.8 (12.02)		*p value*	<.001*
	Married	63.39 (11.61)	**Internal HLC**	Pearson’s rho	.19
	Statistics	t(243) = 2.17		*p value*	.003*
	*p value*	.03*	**External HLC**	Pearson’s rho	.0
**Education**	Secondary or lower	63.21 (11.94)		*p value*	*.99*
	Tertiary	65.55 (11.73)	**Chance HLC**	Pearson’s rho	-.18
	Statistics	t (243) = 1.52		p value	.003*
	*p value*	*.13*	**Sleep quality**	Pearson’s rho	.29
**Working Shifts**	No	64.08 (11.7)		*p value*	*<0.001*
	Yes	65.89 (13.02)			
	Statistics	t(185) = 0.92			
	*p value*	*.36*			
**Smoking**	Yes	59.5 (11.65)			
	No	66.18 (10.96)			
	Statistics	t(201) = 3.99			
	*p value*	<.001*			
**Disease**	Yes	64.71 (12.44)			
	No	64.53 (0.13)			
	Statistics	t(243) = 0.13			
	*p value*	*.9*			

SD: Standard Deviation, BMI: Body Mass Index, PSS: Perceived Stress Scale, HLC: Health Locus of control, HLPCQ: Health Lifestyle and Personal Control Questionnaire.

*Level of significance <0.05.

## Discussion

The aim of this study was to evaluate the psychometric properties of a novel questionnaire that examines several dimensions of daily living. As stated in the background, the items were based on the authors’ experience with collecting qualitative data in the context of conducting stress management programs in different healthy or diseased populations. As such, we intended that this questionnaire, Healthy Lifestyle and Personal Control Questionnaire (HLPCQ), would measure the lifestyle pattern of individuals who have increased control over their health, indicating their degree of empowerment, which is the main goal of contemporary health promotion programs. The PCA analysis resulted in five factors that were named and interpreted and names as follows: 1. Dietary Healthy Choices: representing control over food quantity and quality, 2. Dietary Harm Avoidance: representing control over “food temptations”, such as stress-eating, binge-eating, soft drink and fast-food consumption, 3. Daily Routine: representing the individual’s control over consistent timing of meals and sleep, 4. Organized Physical Exercise: representing the tendency to follow scheduled organized physical exercise and 5. Social and Mental Balance: representing the individual’s inclination to socialize, balance leisure and personal time and adopt positive thinking or cognitive control over stressors. All factors showed satisfactory internal consistency and the scores showed adequate variances relative to the theoretical ranges. All factors were significantly positively related to each other, which indicates that they collectively represent the degree of empowerment and self-efficacy that a person possesses. In the absence of a similar questionnaire, validation was based on measures of perceived stress and health locus of control. As presented in the Results section, perceived stress and internal HLC were significantly correlated with all the aforementioned subscales and the total score, which also indicates that a high HLPCQ score adequately reflects the lifestyle pattern of a self-efficacious, empowered individual. The lack of correlation of HLPCQ with SRRS most likely reflects the inability of an environmental approach of stress (i.e. the occurrence of stressors) to reflect the true stress experienced by the individual (perceived stress), since SRRS obviates the cognitive appraisal of the stressors by the individual [25].

A novel idea of the HLPCQ is Daily Routine (DR). To our knowledge, there is no other instrument that directly assesses routine. The concept of DR derives from the principal regular framework of a day, which certainly consists of eating and sleeping. The words “…at the same time each day”, “…scheduled program” or “…not losing meals” apparently denote a regularity that is crucial for expressing routine. Circadian rhythms are centrally (within the central nervous system) and peripherally (within cells) regulated and stress hormones mediate the overall control [26]. Disruption of daily rhythms is a major stressor that affects physiology and, over time, leads to disease [26]. In contrast, adherence to a daily program reduces stress levels and negative emotions and favors a less reward-seeking behavior (e.g., recreational habits) and more healthy choices on a daily basis [6, 26–28]. A good surrogate marker for the integrity of circadian rhythms is sleep quality [26–28]. Therefore, in support of the validity of our questionnaire, sleep quality was positively correlated with HLPCQ scores and particularly with DR.

Concerning sociodemographic variables, our results indicate that women, unmarried people, non-smokers and normal-weighted individuals have higher HLPCQ scores. Specifically, women score higher for the DHC and SaMB subscales indicating their higher interest in healthy diet, socialization and self-control over their thoughts. These findings agree with previous reports that women are more adherent to healthy diets (“partly attributable to women’s greater weight control involvement and partly to their stronger beliefs in healthy eating” [29]) and that they tend to utilize social support and distraction to cope with stress [30]. Regarding marital status, previous studies show that unmarried people seem to follow a more pro-active lifestyle, although the results of other studies contradict this trend [31–33]. Transitions in marital status (e.g., from marriage to divorce or widowhood or the reverse) and several confounders such as age (unmarried people have poorer health when in older ages) and gender (unmarried men show poorer health) may account for these reported discrepancies [34]. In our study, a majority of unmarried individuals were younger (53.6% and 28.9% were 23–46 and 47–55 years old, respectively, compared to 23.9% and 39.9% for married persons) and more often female (67% of unmarried and 53.5% of married persons were female), which could explain their higher HLPCQ scores compared to married persons. Finally, we believe that our results in smokers and overweight persons indicate reward-seeking behavior (through nicotine or stress eating) that has been confirmed in previous reports [6]. Surprisingly, the presence of disease was not correlated with HLPCQ, although better health assessment did correlate with better scores. One explanation could be that the latter is more intuitive and indicative of someone’s control over health-related activities, while the former does not preclude an empowered individual.

We realize that this study has a number of limitations. First, items were selected based on personal experience and qualitative investigations conducted our team, thus it is possible that some relevant aspects of daily living were not included. Second, no confirmatory factor analysis was performed, which could have further validated our results. Third, the generalization of our results is hampered by our sampling method; participants were not, selected randomly (convenience sample) and did not represent certain regions of Attica. However, our sample was large and quite representative of the adult population, at least with respect to age, gender and education. Finally, due to practical issues no test-retest reliability assessment was performed.

## Conclusions

To summarize, HLPQ is a newly introduced tool that inherently assesses the degree of someone’s control over his/her daily activities in terms of dietary habits, daily program, physical exercise, socialization and negative thoughts. The tool was designed to address health-related daily activities that collectively reflect the degree of empowerment a person has and not to record only healthy lifestyle habits, which is the rule within modern studies. This instrument incorporates a novel idea, Daily Routine that, as described above, reflects biological and physical well-being of the human body. Finally, the questionnaire is short and easily to administer, so we encourage researchers devoted to health promotion to use it. The questionnaire is now available in the Greek language, thus interested researchers (who we will provide them the questionnaire upon request) should follow the same strategy of validation. Further future comparisons with other tools concerning health promotion and psychological measures is strongly encouraged. We hope that the use of the HLPCQ in future research will better explain the efficacy of health promotion interventions by addressing the crucial factors of stress and self-control in terms of actual daily activities, which represent them.

## Authors’ information

Christina Darviri and Evangelos C Alexopoulos shared first authorship.

## References

[1] Weisburger JH (2002). Lifestyle, health and disease prevention: the underlying mechanisms. Eur J Cancer Prev.

[2] Beaglehole R, Yach D (2003). Globalisation and the prevention and control of non-communicable disease: the neglected chronic diseases of adults. Lancet.

[3] WHO (2010). Global Status Report on Non Communicable Diseases.

[4] Noordman J, van der Weijden T, van Dulmen S (2012). Communication-related behavior change techniques used in face-to-face lifestyle interventions in primary care: a systematic review of the literature. Patient Educ Couns.

[5] Capacci S, Mazzocchi M, Shankar B, Macias JB, Verbeke W, Pérez-Cueto FJ, Kozioł-Kozakowska A, Piórecka B, Niedzwiedzka B, D’Addesa D, Saba A, Turrini A, Aschemann-Witzel J, Bech-Larsen T, Strand M, Smillie L, Wills J, Traill WB (2012). Policies to promote healthy eating in Europe: a structured review of policies and their effectiveness. Nutr Rev.

[6] Starcke K, Brand M (2012). Decision making under stress: a selective review. Neurosci Biobehav Rev.

[7] Kalaitzidou I, Venetikou MS, Konstadinidis K, Artemiadis AK, Chrousos G, Darviri C (2013). Stress management and erectile dysfunction: a pilot comparative study. Andrologia.

[8] Christaki E, Kokkinos A, Costarelli V, Alexopoulos EC, Chrousos GP, Darviri C (2013). Stress management can facilitate weight loss in Greek overweight and obese women: a pilot study. J Hum Nutr Diet.

[9] Bougea AM, Spandideas N, Alexopoulos EC, Thomaides T, Chrousos GP, Darviri C (2013). Effect of the emotional freedom technique on perceived stress, quality of life, and cortisol salivary levels in tension-type headache sufferers: a randomized controlled trial. Explore (NY).

[10] Katsarou AL, Vryonis MM, Protogerou AD, Alexopoulos EC, Achimastos A, Papadogiannis D, Chrousos GP, Darviri C (2013). Stress management and dietary counseling in hypertensive patients: a pilot study of additional effect. Prim Health Care Res Dev.

[11] Gika DM, Artemiadis AK, Alexopoulos EC, Darviri C, Chrousos GP, Papanikolaou K (2012). Use of a relaxation technique by mothers of children with autism: a case-series study. Psychol Rep.

[12] Christakis I, Pagkratis MT, Varvogli L, Darviri C, Chroussos G (2012). Measuring the stress of the surgeons in training and use of a novel interventional program to combat it. J Korean Surg Soc.

[13] Artemiadis AK, Vervainioti AA, Alexopoulos EC, Rombos A, Anagnostouli MC, Darviri C (2012). Stress management and multiple sclerosis: a randomized controlled trial. Arch Clin Neuropsychol.

[14] de Ridder DT, Lensvelt-Mulders G, Finkenauer C, Stok FM, Baumeister RF (2012). Taking stock of self-control: a meta-analysis of how trait self-control relates to a wide range of behaviors. Pers Soc Psychol Rev.

[15] Frei A, Svarin A, Steurer-Stey C, Puhan MA (2009). Self-efficacy instruments for patients with chronic diseases suffer from methodological limitations–a systematic review. Health Qual Life Outcomes.

[16] Bakker L, Van Brakel WH (2012). Empowerment assessment tools in people with disabilities in developing countries. A systematic literature review. Lepr Rev.

[17] Darviri C, Fouka G, Gnardellis C, Artemiadis AK, Tigani X, Alexopoulos EC (2012). Determinants of self-rated health in a representative sample of a rural population: a cross-sectional study in Greece. Int J Environ Res Public Health.

[18] Darviri C, Artemiadis AK, Tigani X, Alexopoulos EC (2011). Lifestyle and self-rated health: a cross-sectional study of 3,601 citizens of Athens, Greece. BMC Public Health.

[19] Cohen S, Mermelstein K, Kamarck T (1983). A global measure of perceived stress. J Health Soc Behav.

[20] Andreou E, Alexopoulos EC, Lionis C, Varvogli L, Gnardellis C, Chrousos GP, Darviri C (2011). Perceived stress scale: reliability and validity study in Greece. Int J Environ Res.

[21] Masuda M, Holmes TH (1967). Magnitude estimations of social readjustments. J Psychosom Res.

[22] Holmes TH, Rahe RH (1967). The social readjustment rating scale. J Psychosom Res.

[23] Wallston KA, Wallston BS, DeVellis R (1978). Development of the multidimensional health locus of control scale. Health Educ Monogr.

[24] Karademas EC (2009). Effects of exposure to the suffering of unknown persons on health-related cognitions, and the role of mood. Health (London).

[25] Cohen S, Kessler RC, Gordon LU (1997). Measuring Stress: A Guide for Health and Social Scientists.

[26] Kino T, Chrousos GP (2011). Circadian CLOCK-mediated regulation of target-tissue sensitivity to glucocorticoids: implications for cardiometabolic diseases. Endocr Dev.

[27] Lightman SL (2008). The neuroendocrinology of stress: a never ending story. J Neuroendocrinol.

[28] Buckley TM, Schatzberg AF (2005). On the interactions of the hypothalamic-pituitary-adrenal (HPA) axis and sleep: normal HPA axis activity and circadian rhythm, exemplary sleep disorders. J Clin Endocrinol Metab.

[29] Wardle J, Haase AM, Steptoe A, Nillapun M, Jonwutiwes K, Bellisle F (2004). Gender differences in food choice: the contribution of health beliefs and dieting. Ann Behav Med.

[30] Rao K (2009). Recent research in stress, coping and women’s health. Curr Opin Psychiatry.

[31] Ortega FB, Brown WJ, Lee DC, Baruth M, Sui X, Blair SN (2011). In fitness and health? A prospective study of changes in marital status and fitness in men and women. Am J Epidemiol.

[32] Yim HJ, Park HA, Kang JH, Kim KW, Cho YG, Hur YI, Choi OJ (2012). Marital status and health behavior in middle-aged Korean adults. Korean J Fam Med.

[33] Joung IM, Stronks K, van de Mheen H, Mackenbach JP (1995). Health behaviours explain part of the differences in self reported health associated with partner/marital status in The Netherlands. J Epidemiol Community Health.

[34] Robards J, Evandrou M, Falkingham J, Vlachantoni A (2012). Marital status, health and mortality. Maturitas.

[CR35] The pre-publication history for this paper can be accessed here:http://www.biomedcentral.com/1471-2458/14/995/prepub

